# The Role of Electronic Medical Record Automation in Latent Tuberculosis Screening and Treatment in a Large Health System

**DOI:** 10.1093/ofid/ofaf582

**Published:** 2025-09-22

**Authors:** Hannah Li, Andrea Polesky, Andrea Cervenka, Harleen Sahni

**Affiliations:** Internal Medicine, Santa Clara Valley Medical System, San Jose, California, USA; Infectious Diseases, Santa Clara Valley Medical System, San Jose, California, USA; Internal Medicine, Santa Clara Valley Medical System, San Jose, California, USA; Infectious Diseases, Santa Clara Valley Medical System, San Jose, California, USA

**Keywords:** electronic medical record automation, latent tuberculosis, tuberculosis screening

## Abstract

After an electronic medical record tool was implemented in primary care, tuberculosis screening increased from 6202 to 16 394 patients. There was an absolute increase in, but not a higher percentage of, patients starting and completing preventative treatment. This low-cost intervention highlights the electronic medical record's potential to enhance tuberculosis prevention.

The World Health Organization estimates that a quarter of the world's population has been infected with tuberculosis (TB) [1]. While overall latent tuberculosis infection (LTBI) prevalence in the United States (US) is low, interferon-gamma release assay (IGRA) positivity was 15.9% among non-US-born persons during the 2011–2012 National Health and Nutritional Examination Survey [2]. In 2022, 73% of active US TB cases were among foreign-born persons and it is estimated that most of these were due to progression of LTBI rather than new TB transmission [3, 4]. The United States Preventative Services Task Force recommends LTBI screening for asymptomatic adults at higher risk, including those born or residing in TB-endemic countries or living in high-risk congregate settings [5]. Mathematical modeling of TB transmission shows that increasing LTBI treatment of foreign-born patients will be necessary to approach US goals for TB elimination [6].

Santa Clara County had the second highest TB rate in California in 2023 and an estimated LTBI prevalence of 8.5% (see Supplementary material). In 2023, 97% of TB cases in the county occurred among non-US-born persons, with 71% having lived in the US for at least 10 years (see Supplementary material). Recent Kaiser Permanente California studies found that birth in a TB-endemic country was the strongest risk factor for identifying incident TB cases [7], yet non-US-born persons had lower screening rates than other risk groups [8]. While Santa Clara Valley Health (SCVH) System clinics routinely screen patients with or about to undergo immunosuppression, refugees, new immigrants with abnormal chest films, persons experiencing homelessness, and persons in the jail system, non-US-born persons outside of these groups are not systematically screened.

The LTBI care cascade is a public health model that follows patients through the multiple steps from TB screening to LTBI treatment completion. A Centers for Disease Control and Prevention survey found that only 53.3% of healthcare providers routinely test non-US-born persons for LTBI [3]. A University of Washington study found that only 20% of eligible patients were screened [9]. Through a collaboration between infectious diseases, adult primary care, and health informatics, we utilized the electronic medical record (EMR) to improve LTBI screening by implementing an automated prompt for physicians to order an IGRA in patients with specific risk factors, including primary language other than English (PLOE) as a proxy for foreign birth, to increase screening and treatment among non-US born adults.

## METHODS

### Study Sites

This study included 8 clinics (East Valley, Downtown San Jose, Gilroy, Milpitas, Moorpark, San Jose, Sunnyvale, and Tully) within the SCVH System that provided most of the primary care between 2021 and 2023. The Tuberculosis and Refugee Health Clinic, a specialty SCVH clinic, oversees multiple LTBI clinics, on-site and at 4 embedded Valley Health Center (VHC) locations: Gilroy, Downtown, Tully, and Milpitas.

### Study Interventions

Within EPIC Systems, an EMR tool was developed to prompt physicians to order an IGRA for patients aged 18 years or older who have no documented prior TB screening test and who meet one of the following criteria: Human immunodeficiency virus (HIV) infection, a substance use diagnosis (SUD), a history of homelessness, PLOE, or presence within the Santa Clara County jail system. PLOE was used as a proxy for foreign birth, since patients' country of birth is not routinely documented in the EMR's demographics section. If a patient meets screening criteria, an alert appears in their chart under “Current Care Gaps” reminding the physician to order an IGRA. If an IGRA is performed the alert disappears. The EMR “Care Gap” for IGRA screening went live on 9 August 2022.

### Data Collection and Analysis

We used the EMR data-collection tool Slicer Dicer to determine the total adult primary care population at VHC clinics with visits (office, telehealth) and, within this group, patients with a PLOE, living with HIV (PLWH), or with a SUD. For these subgroups, we determined the number of IGRAs obtained and the number of positive IGRAs during the periods 8/9/21–8/8/22 and 8/9/22–8/8/23. We analyzed data from our institution's LTBI clinics using an EMR data base that tracks LTBI initiation and completion rates. The percentage of PLWH with a history of TB testing was determined using an EMR database report from the Partners in AIDS Care and Education (PACE) clinic, a specialty clinic for HIV care.

Statistical analysis was performed using R statistical software. We used a z-score for 2 population proportions to analyze the differences between the 2 groups.

### Ethical Approval

The Institutional Review Board determined that this project does not meet the Federal Regulations definition of research and did not require approval.

## RESULTS

Between 8/9/21–8/8/22, there were 62 432 patients with a VHC primary care clinic visit of which 25 128 patients (40.2%) spoke a PLOE. From 8/9/22–8/8/23, patients with a PLOE made up 28 145 of 67 657 (41.6%) patients with a visit.

In the year after implementation of the EMR tool, the number of patients with an IGRA performed increased from 6202 to 16 394. Among patients with a PLOE, the number tested increased by 450% (2448 to 11 024), compared to a 43% increase among primary English speakers (3754 to 5370). The proportion of positive IGRA results increased from 10.2% to 14.2% among the primary care population, and from 15.3% to 18.0% among those with a PLOE (Table 1). The proportion of IGRAs performed in patients with a PLOE increased from 39.5% (2448/6202) to 67.2% (11 024/16 394), and the proportion of positive IGRAs in patients with a PLOE increased from 59.3% (375/632) to 85.3% (1981/2323).

**Table 1.** LTBI Care Cascade Before and After Implementation of the Tuberculosis Screening “Care Gap” Reminder.

	Year Before Implementation^a^	Year After Implementation	
	Patients, Number (%)		*P*-value
Total Primary Care Population^b^	62 432	67 657	…
IGRA Performed	6202/62 432 (9.9)	16 394/67 657 (24.2)	<.001
IGRA Positive	632/6202 (10.2)	2323/16 394 (14.2)	<.001
Primary Language Other Than English Population^c^	25 128	28 145	…
IGRA Performed	2448/25 128 (9.7)	11 024/28 145 (39.2)	<.001
IGRA Positive	375/2448 (15.3)	1981/11 024 (18.0)	<.01
Patients with a Substance Use Diagnosis^d^	3598	3921	…
IGRA Performed	402/3598 (11.2)	1027/3921 (26.2)	<.001
IGRA Positive	34/402 (8.5)	83/1027 (8.1)	.8
Patients Living With HIV^e^	1097	1231	…
IGRA Performed	478/1097 (43.6)	555/1231 (45.1)	.5
IGRA Positive	25/478 (5.2)	28/555 (5.0)	.9
Data from LTBI Clinics^f^	Patients, Number (%)		*P*-value
Treatment Initiated	291	752	…
Treatment Completed	204/291 (70.1)	533/752 (70.9)	.8

Abbreviations: LTBI, latent tuberculosis infection; IGRA, interferon gamma release assay; HIV, Human Immunodeficiency Virus.

^a^The “Care Gap” reminder was implemented on 9 August 2022.

^b^This is the total number of patients age ≥ 18 with an office or telehealth visit at the Valley Health Care clinics.

^c^This is the total number of patients age ≥ 18 with an office or telehealth visit at the Valley Health Care clinics whose primary language was not English. Primary language other than English was used as a proxy for birth outside of the United States.

^d^This is the total number of patients age ≥ 18 with an office or telehealth visit at the Valley Health Care clinics who have a substance use diagnosis.

^e^This is the total number of patients age ≥ 18 living with HIV with an office or telehealth visit at the Valley Health Care clinics.

^f^This is the number of patients age ≥ 18 referred from the Santa Clara Valley Health System to the LTBI Clinics who initiated and completed treatment.

After implementation of the EMR tool, the number of IGRA's performed among patients with a SUD increased from 11.2% (402/3598) to 26.2% (1027/3921). In PLWH with primary care visits, the number of IGRAs performed were similar during the 2 time periods, 43.6% (478/1097) and 45.1% (555/1231) (Table 1). Within the HIV specialty clinic, a TB screening test result was available for 95.7% (1386/1449) of patients between 8/9/21–8/8/22 and 96.2% (1547/1608) between 8/9/22–8/8/23. IGRA positivity was similar for both populations before and after implementation of the “Care Gap”.

In the year before the “Care Gap” was implemented, 291 of 632 (45.9%) patients from the SCVH System initiated LTBI treatment and 204 patients (70.1%) completed treatment within our institution's LTBI clinics. In the following year, 752 of 2323 (32.4%) patients initiated treatment and 533 patients (70.9%) completed treatment (Table 1).

## DISCUSSION

This study shows that a low-cost EMR intervention can substantially increase LTBI screening in primary care settings. In the year after implementation, the number of IGRAs performed increased from 9.9% to 24.2% (6202 to 16 394). PLOE patients made up a larger portion of the tested population, increasing from 39.5% to 67.2%. The greater proportion of positive IGRAs among PLOE after the intervention (18.0%) is interesting and may reflect expanded testing of healthier adults not routinely screened prior. By instituting an EMR “Care Gap” reminder and systematically including PLOE as a proxy for foreign birth, we identified a large group of IGRA-positive patients missed by prior screening programs.

Testing also increased among patients with a SUD. However, testing among PLWH was stable, reflecting the longstanding TB screening program in the specialty clinic for PLWH, where more than 95% of patients had documented TB test results before and after the intervention.

Although targeting the first step of the LTBI cascade was successful, success at 1 step does not ensure success at subsequent steps. Consistent with other LTBI care cascade studies, there was a significant drop-off from positive IGRA to treatment initiation [9, 10]; despite more than 2.5 times as many patients initiating and completing treatment, the overall proportion of IGRA-positive patients treated did not increase. This study did not explore reasons for this, such as failure to obtain a chest X-ray, prior evaluation and treatment, lack of referral, missed appointments, medical contraindications, and treatment refusal. Additionally, IGRA positive patients identified towards the end of the post-intervention year may have been evaluated and treated after the study period.

A limitation of the study was that not all indications for TB screening related to immunosuppression were included due to the complexity of programming the EMR. However, the large PLOE population, for whom no screening program existed, and other high-risk groups were included. Slicer Dicer also lacked variables to analyze homeless or jail populations. We could not determine the total eligible screening population, since EMR prompts are not trackable once resolved. Thus, we chose to assess overall changes in screening relative to total primary care populations.

A further limitation is that since PLOE was used as a proxy for non-US birth, foreign-born patients with English listed as the primary language are missed. We also recognize that screening likely increased as the COVID-19 pandemic subsided, however the large relative increases in screening of two of the targeted populations cannot be explained by this alone.

Finally, the number of patients treated in our institution's LTBI clinics may not reflect all treated patients. We were unable to assess whether LTBI patients were treated in other settings. However, primary care providers in our system typically refer patients to the LTBI clinics, and we do not believe referral patterns changed during the study period. The treatment count also includes some individuals referred from the hospital and subspecialty clinics.

This study demonstrates that EMR automation prompting TB testing for targeted populations is effective. Including PLOE as a prompt identified a large patient group with a high proportion of positive IGRAs. However, retaining patients on the LTBI care cascade is complex and resource intensive requiring continuous patient and provider education, reduction in barriers to care, and dedicated patient tracking and follow up. Our next step is to identify where the greatest loss of eligible patients occurs and leverage the power of the EMR as a resource-efficient way to improve their retention in care.

## Supplementary Material

ofaf582_Supplementary_Data

## Supplementary Data

Supplementary materials are available at *Open Forum Infectious Diseases* online. Consisting of data provided by the authors to benefit the reader, the posted materials are not copyedited and are the sole responsibility of the authors, so questions or comments should be addressed to the corresponding author.
