# Supplementary material for: The Role of Electronic Medical Record Automation in Latent Tuberculosis Screening and Treatment in a Large Health System
Source: Open Forum Infect Dis. 2025 Sep 22;12(10):ofaf582. doi: 10.1093/ofid/ofaf582 (PMC12502649; doi:10.1093/ofid/ofaf582)
Supplement: ofaf582_Supplementary_Data [file ofaf582_supplementary_data.pdf]

Tuberculosis in Santa Clara County, 2023

Santa Clara County PUBLIC HEALTH

About us

The Santa Clara County Public Health Department Tuberculosis (TB) Prevention & Control Program investigates all reports of persons with confirmed or suspected TB disease in Santa Clara County. We provide individualized case management to each patient to help ensure treatment completion and to prevent TB from spreading further. We work with Civil Surgeons to identify and treat latent TB infection (LTBI), and we provide consultation to medical providers and community organizations, thus creating partnerships for the prevention of tuberculosis.

TB Summary

TB is a preventable and curable communicable disease. TB is caused by the bacteria *Mycobacterium tuberculosis*, which can spread from person to person through the air when an individual with infectious TB disease coughs, sneezes, or speaks. Transmission occurs when others breathe in the bacteria while in close and prolonged contact with a person with infectious TB disease. TB bacteria can infect anyone regardless of their age, race, sex, or socioeconomic status.

LTBI occurs when individuals are infected with the bacteria that causes TB. In contrast with TB disease or "active TB" individuals with LTBI are not contagious. If, over the course of months to years, the body's immune system can no longer control the latent infection, the bacteria multiply and cause TB disease. The risk of developing TB disease after infection is higher for folks with any condition that impairs the body's ability to control the infection, such as being underweight, having HIV infection, taking immunosuppressing treatment, having diabetes, etc. [1]. Certain behaviors, such as alcohol use and smoking, also increase an individual's risk for developing TB disease. If treatment is delayed, TB disease can cause serious illness and death. Fortunately, TB can be prevented, treated, and cured!

TB Cases and Rates

There were 168 cases of TB disease in Santa Clara County (SCC) in 2023 which is a 19% increase compared to 2022 (141 TB cases) (Figure 1). This represents a case rate of 8.8 per 100,000 people. This case rate is 1.6 times as high as the 2023 overall California rate (5.4) (Figure 2) and 3.0 times as high as the national rate (2.5) [2,3]. In 2023, SCC had the second highest case rate among all California jurisdictions, after Imperial County [2].

Figure 1: Trends in TB Case Counts and Rates in Santa Clara County, 2010-2023

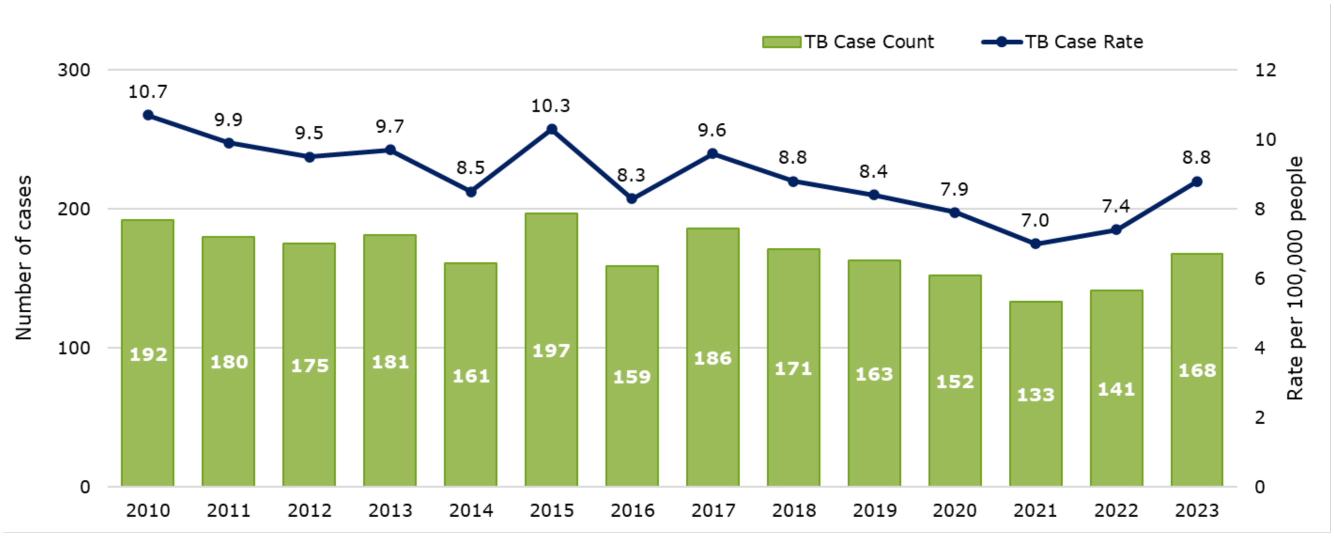

Source: California Reportable Disease Information Exchange, 2010-2023. Data as of January 30, 2024, and are provisional; State of California, Department of Finance, Race/Ethnic Population with Age and Sex Detail, 2010-2060. Sacramento, California, July 2021; State of California, Department of Finance, E-2. California County Population Estimates and Components of Change by Year — July 1, 2010–2021, December 2021; State of California, Department of Finance, E-2. California County Population Estimates and Components of Change by Year — July 1, 2020-2023, December 2023.

Figure 2: TB Case Rates for California and San Francisco Bay Area Counties, 2023

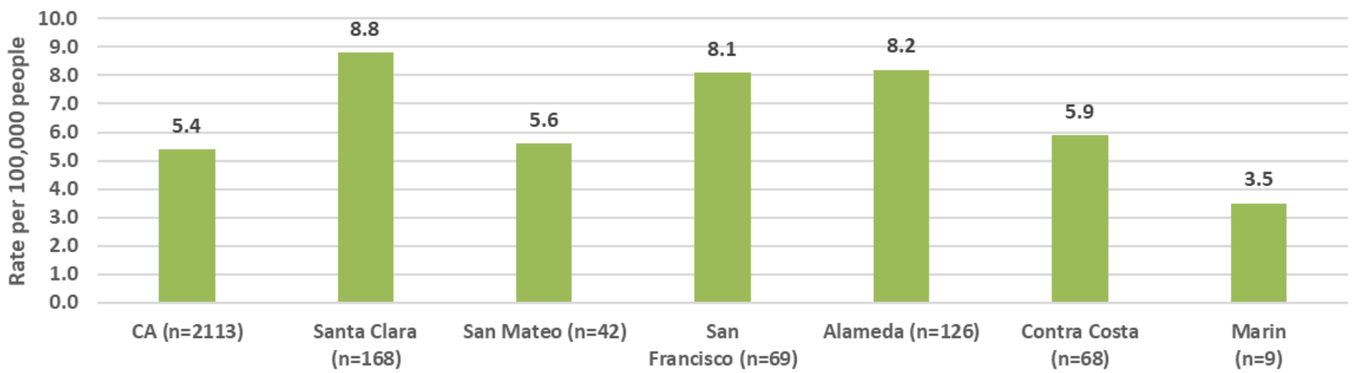

Source: California Department of Public Health, TB Control Branch.

Medical Comorbidities

In 2023, 36% of people with TB in SCC had one or more medical conditions associated with an increased risk of progression from latent tuberculosis infection to TB disease. The most common comorbidity in 2023 was diabetes mellitus (26%). Other risk factors included immunosuppression not related to HIV/AIDS (11%), alcohol use (1%), drug use (1%), and HIV infection (1%).

TB Cases by Race/Ethnicity

TB cases in SCC during 2023 occurred predominantly among Asian (76%) and Hispanic (19%) populations, with a small percentage in African American/African Ancestry (2%), White (1%) and Native Hawaiian or other Pacific Islander (1%) populations (Figure 3).

Figure 3: TB Cases by Race/Ethnicity in Santa Clara County, 2023

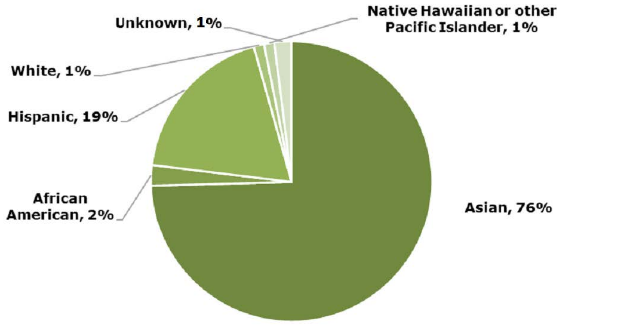

Source: Santa Clara County Public Health Department, California Reportable Disease Information Exchange, 2024. Data as of January 30, 2024, and are provisional.

In 2023, about 97% of TB cases occurred among persons born outside the U.S., primarily from the following countries: Vietnam (26%), the Philippines (21%), India (17%), Mexico (14%) and China (6%). Case rates by country of origin was highest among those born in the Philippines (65.0 per 100,000 people) and Vietnam (44.2) followed by India (20.6), Mexico (19.1), which are much higher than the SCC overall case rate (8.8) (Figure 4). The case rate among those born in China was 7.0.

Length of Time in US

In 2023, about 17% of persons with TB in SCC who were born outside the U.S. had lived in the U.S. for less than 5 years. Most persons with TB (71%) had lived in the U.S. for at least 10 years.

Figure 4: TB Rates by Country of Birth in Santa Clara County, 2023

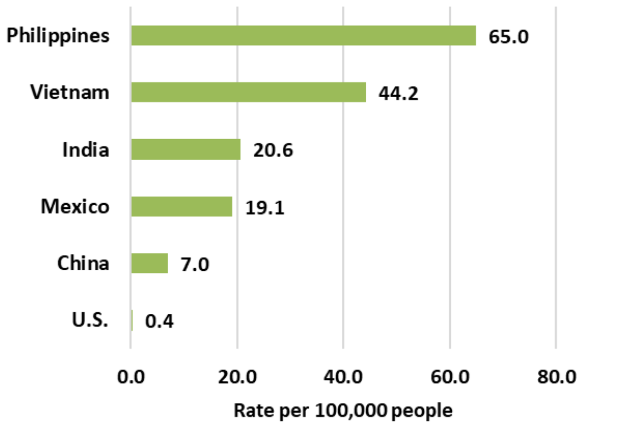

Source: Santa Clara County Public Health Department, California Reportable Disease Information Exchange, 2024. Data as of January 30, 2024, and are provisional; U.S. Census Bureau; 2018-2022 American Community Survey 5-Year Estimates, Table B05006, Table DP02; generated by Public Health Science Branch; using data.census.gov; Accessed 04032024.; State of California, Department of Finance, E-2. California County Population Estimates and Components of Change by Year – July 1, 2020-2023, December 2023.

Age Group

In 2023, people over 65 years old accounted for most TB cases (48%), followed by people 45-64 years old (25%), then 25-44 years old (21%). People less than 25 years old accounted for 6% of TB cases.

TB Drug Resistance

Isoniazid Resistance

Among people born outside the U.S. with culture-positive TB during 2010–2023, isoniazid (INH) resistance was present in 13% of those who had no prior history of TB and 19% of those with a prior history of TB. In 2022, in the US as a whole 8% of TB cases were resistant to INH. Among people with culture-positive TB and no prior history of TB, isoniazid resistance rates were highest among those born in Vietnam (18%), the Philippines (16%), and India (9%) (Figure 5).

Figure 5: Percentage of INH Resistance per Country of Birth — Santa Clara County, 2010–2023

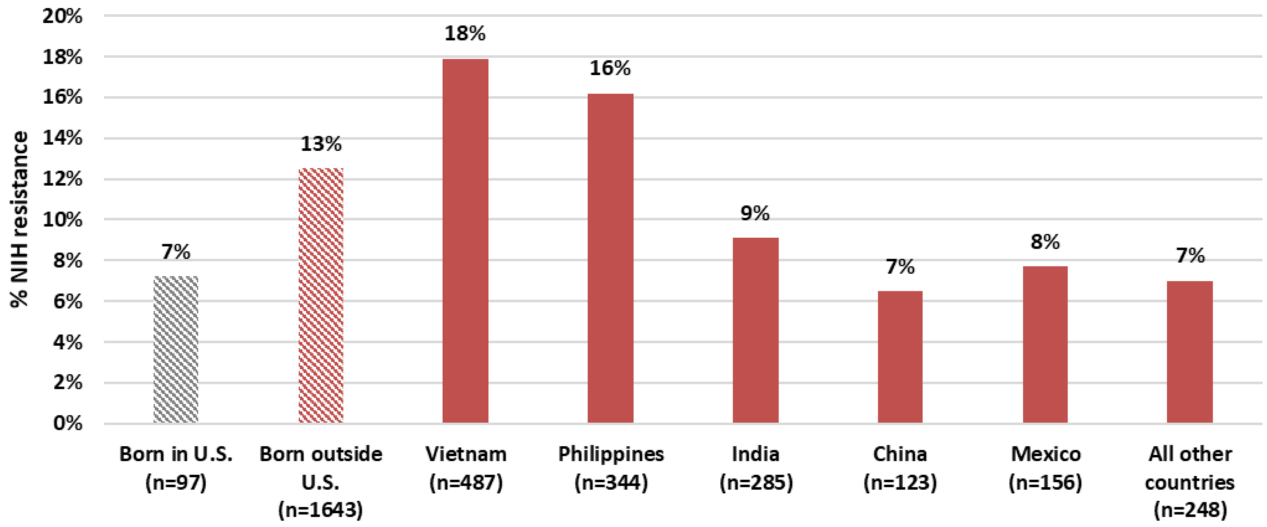

Cases are culture-positive with initial drug susceptibility testing done and no prior history of TB. Excludes cases with susceptibility testing not done or unknown for isoniazid. Source: Santa Clara County Public Health Department, California Reportable Disease Information Exchange, 2010-2023; Data as of January 30, 2024

Multidrug-Resistant (MDR), pre-Extensively Drug-Resistant (pre-XDR), and XDR TB

Definition for TB drug resistance were updated in 2022 [4]. MDR-TB cases are resistant to both isoniazid and rifampin; pre-XDR cases are additionally resistant to a fluoroquinolone or a second-line injectable. XDR cases are resistant to isoniazid, rifampin, and at least one fluoroquinolone, and either a second-line injectable OR bedaquiline OR linezolid. Using these new definitions, from 2010–2023 there have been 36 people diagnosed with MDR-TB in SCC: 39% were born in India, 31% were born in Vietnam, 8% were born in the Philippines, 6% were born in the U.S., and 3% were born in Mexico. Since 2010, there have been seven pre-XDR cases and no known XDR cases in SCC. There were two MDR-TB cases, one pre-XDR, and no XDR cases in 2023.

Rapid Molecular Testing to Detect Mycobacterium tuberculosis and Multi-drug Resistance

CDC recommends the use of a rapid molecular test on at least one specimen from each patient with signs and symptoms of pulmonary tuberculosis for whom a diagnosis of tuberculosis is being considered but has not been established [5]. Use of molecular tests directly on clinical samples has been shown to shorten time to diagnosis, and some tests have the additional ability to provide information on drug susceptibility or provide an early indication of possible rifampin resistance (e.g. Xpert MTB/RIF) [5,6,7]. As most people with rifampin resistance have multi-drug resistance, this information can expedite initiation of an appropriate treatment regimen in consultation with the Public Health Department. The Xpert MTB/RIF assay is available

through the Santa Clara County Public Health Laboratory and many regional labs. In 2023, NAATs were used for 89% of pulmonary TB cases in SCC.

Global Perspective

The World Health Organization (WHO) estimates that about one out of four people in the world, or 2 billion people, are infected with *Mycobacterium tuberculosis* [1]. An estimated 10.6 million people developed TB disease and 1.3 million people died of TB disease in 2022. Most cases of TB disease occurred in South-East Asia (46%), Africa (23%), and the Western Pacific (18%). An estimated 6.3% of incident TB cases had HIV co-infection. Among incident TB cases, about 3.3% were estimated to have multidrug-resistant TB. TB treatment coverage was 70% in 2022, higher than 62% in 2021 and 58% in 2020.

Prevention

The California Department of Public Health estimates that in Santa Clara County, about 170,000 people have latent TB infection (LTBI) [8]. This represents a very large reservoir of individuals from which future cases of TB disease will develop. In order to significantly decrease the number of people with TB disease, more individuals with risk factors for TB need to be tested and treated for LTBI. This underscores the need for primary care providers to conduct targeted testing and treatment for LTBI as part of routine preventive care. Treatment for LTBI is very effective – it can decrease the risk of developing TB disease by over 90% when medications are taken as prescribed [6]. Short-course regimens (i.e. isoniazid-rifapentine, which is given weekly for 12 weeks, rifampin, which is given daily for 4 months, or isoniazid plus rifampin, which is given daily for 3 months) are preferred (except in persons for whom there is a contraindication, such as a drug interaction or contact to a person with drug-resistant TB) due to similar efficacy and higher treatment completion rates as compared with isoniazid given daily for 9 months [6,9,10,11,12].

References

1. World Health Organization. *Global Tuberculosis Report 2023*.
2. California Department of Public Health, Tuberculosis Control Branch. *World TB Day Provisional TB Data Tables, 2023*.
3. Schildknecht K.R., et al. *Tuberculosis – United States, 2022*. MMWR, 2023; 72(12): 297–303.
4. Division of Tuberculosis Elimination, National Center for HIV, Viral Hepatitis, STD, and TB Prevention, Centers for Disease Control and Prevention. *Surveillance definitions for extensively drug resistant (XDR) and pre-XDR tuberculosis*. January 18, 2022.
5. Lewinsohn DM, et al. *Official American Thoracic Society/Infectious Diseases Society of America/Centers for Disease Control and Prevention Clinical Practice Guidelines: Diagnosis of Tuberculosis in Adults and Children*. Clinical Infectious Diseases. 2017; 64(2): 1-33.
6. Guidelines for the Treatment of Latent Tuberculosis Infection: *Recommendations from the National Tuberculosis Controllers Association and CDC, 2020*
7. Boehme CC, Nicol MP, Nabeta P, et al. *Feasibility, diagnostic accuracy, and effectiveness of decentralised use of the Xpert MTB/RIF test for diagnosis of tuberculosis and multidrug resistance: a multicentre implementation study*. Lancet 2011; 377: 1495–505.
8. California Department of Public Health, Tuberculosis Control Branch. *California Tuberculosis Data Tables, 2020.*
9. U.S. Preventive Services Task Force. *Screening for Latent Tuberculosis Infection in Adults: US Preventive Services Task Force Recommendation Statement*. JAMA. 2016; 316: 962-969.
10. Sterling TR, Villarino ME, Borisov AS, et al. *Three months of rifapentine and isoniazid for latent tuberculosis infection.* N Engl J Med. 2011; 365: 2155-66.
11. Menzies D, Adjobimey M, Ruslami R, et al. *Four Months of Rifampin or Nine Months of Isoniazid for Latent Tuberculosis in Adults.* N Engl J Med. 2018; 379: 440-453.
12. Centers for Disease Control and Prevention. *Update of Recommendations for Use of Once-Weekly Isoniazid-Rifapentine Regimen to Treat Latent Mycobacterium tuberculosis Infection.* MMWR. 2018; 67: 723-726.

Additional Resources

- SCC Public Health Department - Residents: www.sccphd.org/tbinfo; Providers: www.sccphd.org/tb
- Centers for Disease Control and Prevention TB resources: https://www.cdc.gov/tb/
- California Department of Public Health Tuberculosis Control Branch: https://www.cdph.ca.gov/Programs/CID/DCDC/Pages/TBCB.aspx
- Curry International Tuberculosis Center: http://www.currytbcenter.ucsf.edu
- California Tuberculosis Controllers Association (CTCA): http://www.ctca.org

Santa Clara County Public Health Department
Tuberculosis Prevention and Control Program
976 Lenzen Ave, Ste. 1700, San Jose, CA 95126
Phone: (408) 792-1381, Fax: (408) 855-2331
WWW.SCCPHD.ORG
